# Three-Year Retrospective Comparative Study between Implants with Same Body-Design but Different Crest Module Configurations

**DOI:** 10.3390/dj8040135

**Published:** 2020-12-15

**Authors:** Silvio Mario Meloni, Luca Melis, Erta Xhanari, Marco Tallarico, Giovanni Spano, Milena Pisano, Edoardo Baldoni, Gabriele Cervino, Antonio Tullio, Aurea Immacolata Lumbau

**Affiliations:** 1 School of Dentistry, University of Sassari, 07100 Sardinia, Italy; melonisilviomario@yahoo.it (S.M.M.); giovanni.spano@aousassari.it (G.S.); baldoni@uniss.it (E.B.); alumbau@uniss.it (A.I.L.); 2 Private Practice, 07100 Sardinia, Italy; lucamelis.od@gmail.com (L.M.); milenapisano@yahoo.it (M.P.); 3 Dentistry Program, Aldent University, 1022 Tirana, Albania; info@ertaxhanari.com; 4 Dental School, University of Messina, 98100 Messina, Italy; gcervino@unime.it; 5 School of Dentistry, University of Perugia, 06123 Perugia, Italy; atullio@unipg.it

**Keywords:** dental implants, implant–abutment connection, marginal bone loss, machined collar, implant depth

## Abstract

Crest module can be defined as the portion of a two-piece implant designed to retain the prosthetic components and to allows the maintenance of the peri-implant tissues in the transition zone. Aim: To evaluate the three-year after loading clinical and radiographic data, collected from patients that received a prosthetic rehabilitation on conical connection implants with partial machined collar (PMC; CC Group) and same body-designed implants, with flat-to-flat connection and groovy neck design (FC Group). Materials and Methods: A retrospective chart review of previously collected data, including documents, radiographs, and pictures of patients who received at least one implant-supported restoration on NobelReplace CC PMC or NobelReplace Tapered Groovy implants was performed. Patients with at least three years of follow-up after final loading were considered for this study. Outcomes measures were implant and prosthesis failures, any biological or technical complications, marginal bone loss. Results: Eight-two patients (44 women, 38 men; average age 55.6) with 152 implants were selected and divided in two groups with 77 (CC group) and 75 (FC group), respectively. Three years after final loading, one implant in CC group failed (98.7% survival rate), while no implants failed in FC group (100% survival rate). One restoration failed in CC group (98.7% survival rate) with no restoration failing in the FC one (100% survival rate). Differences were not statistically significant (*p* = 1.0). Three years after final loading, mean marginal bone loss was 0.22 ± 0.06 mm (95% CI 0.2–0.24) in CC group and 0.62 ± 0.30 mm (95% CI 0.52–0.72) in FC group. The difference was statistically significant (0.40 ± 0.13 mm; 95% CI 0.3–0.5; *p* = 0.003). Conclusion: with the limitation of this retrospective comparative study, implants with conical connection and partial machined collar seem to achieve a trend of superior outcomes if compared with implants with flat connection and groovy collar design.

## 1. Introduction

As shown by several studies, the peri-implant bone loss (or Marginal Bone Loss, MBL) that occurs after prosthetic connection is an important criterion for implant survival predictability [1,2,3,4]. In 1981, Adell and co-authors reported that the bone loss during the first year since abutment connection was 1.2 mm, with an additional annual vertical bone loss of approximately 0.2 mm [5]. Subsequently, Albrektsson described a success implant, a dental implant if the MBL in the first year is less than 1.5 mm, with a subsequent annual MBL of 0.2 mm [3]. More recent studies, however, argue that MBL after prosthetic connection should be as low as possible, with a tolerance that should not exceed 0.5 mm after three years [6] or 5 years [7] of observation.

Achieving a reduction of the MBL continues to be one of the challenging targets for clinicians and researchers [8]. Bone remodeling starts during tissue healing at the implant–abutment connection [9], however, the etiology of these changes has not yet been fully explained and is still object of several investigations [10,11].

Across the years, several factors have been associated with the MBL [10,11,12,13,14,15,16,17], such as gingival biotype and thickness [12,13], surgical trauma [3,10,11], implant micro- and macro-morphology [1,11], type of crestal module (including the platform switching (PS) concept) [1,2], kind of pathogenic flora established at the microgap level within implant–abutment connection, biomechanical stress due to occlusal loading [14], inter-implant distance, thin buccal wall, implant depth position [16], type of implant–abutment connection [17], biological width, and bone density. Some studies have also observed that two-piece implants could lead to formation of a thicker biological width [18], specifically in its connective tissue component. Some other factors seem not be related to the marginal bone level changes as: one-stage rather than two-stage surgery or tilted rather than axial implant placement [19].

Crest module can be defined as the portion of a two-piece dental implant designed to retain the prosthetic components and to allows for the establishment and maintenance of the peri-implant tissues in the transition zone. Several strategies have been described to potentially achieve zero bone loss after implant-prosthetic treatment. Since initial studies, many observations have found that soft and hard tissues seem to respond more favorably to implants connected with platform switching concept [20,21,22,23,24,25,26,27]. However, controversies about the long-term validity of PS system still exist. Moreover, independently by the implant–abutment configuration, limiting the number of times the implant abutment is removed and re-applied could be beneficial to reduce the amount of marginal bone remodeling [28].

The aim of this retrospective comparative study was to evaluate data collected from patient who underwent to a prosthetic rehabilitation with conical connection implants with partial machined collar (CC Group) and flat connection implants with groovy collar (FC Group) after a three-year follow-up since the moment of final loading. This study follows the Strengthening the Reporting of Observational Studies in Epidemiology (STROBE) guidelines for cohort studies.

## 2. Materials and Methods

This study was designed as a retrospettive comparative study. A cohort of patients who received at least one NobelReplace CC PMC and/or one NobelReplace Tapered Groovy implant placed between March 2003 and December 2013 was considered. Patients were grouped based on their exposure to the different implant neck design: conical connection implants with partial machined collar (CC Group), and flat-to-flat connection with groovy neck design (FC Group). A retrospective chart review of previously collected data, including documents, radiographs, and pictures, was performed by an independent examiner. All surgical procedures were performed by the same oral surgeon. All the restorations were performed by the same dentist and dental technician. This study was conducted according to the principles embodied in the Helsinki Declaration of 2013. Patients were informed about clinical procedures, materials to be used, benefits, potential risks and complications, and their written informed consent was obtained for the performed procedures. Medical data was anonymized so that patient cannot be identified. The study protocol was approved by the Ethics Committee of Aldent University in Tirana (Protocol n° 2/2020, 3 April 2020). Any patients aged 18 years or older, who received at least one implant-supported restoration on tested implants, with at least three years of follow-up after final loading, with available radiographs and signed informed consent, were considered for this study. Any potential implant location and prosthetic treatment option were considered in the present research. In the case of patients needing guided bone regeneration or sinus augmentation, the implant sites had to heal for at least 6 months. The exclusion criteria were general contraindications for oral surgery, heavy smokers (≥11 cigarettes per day), post-extractive implants, local acute or chronic infections, poor oral hygiene (bleeding on probing and/or plaque index > 25%), absence of teeth in the opposite jaw, and severe bruxism or jaw clenching.

**Figure 1:**
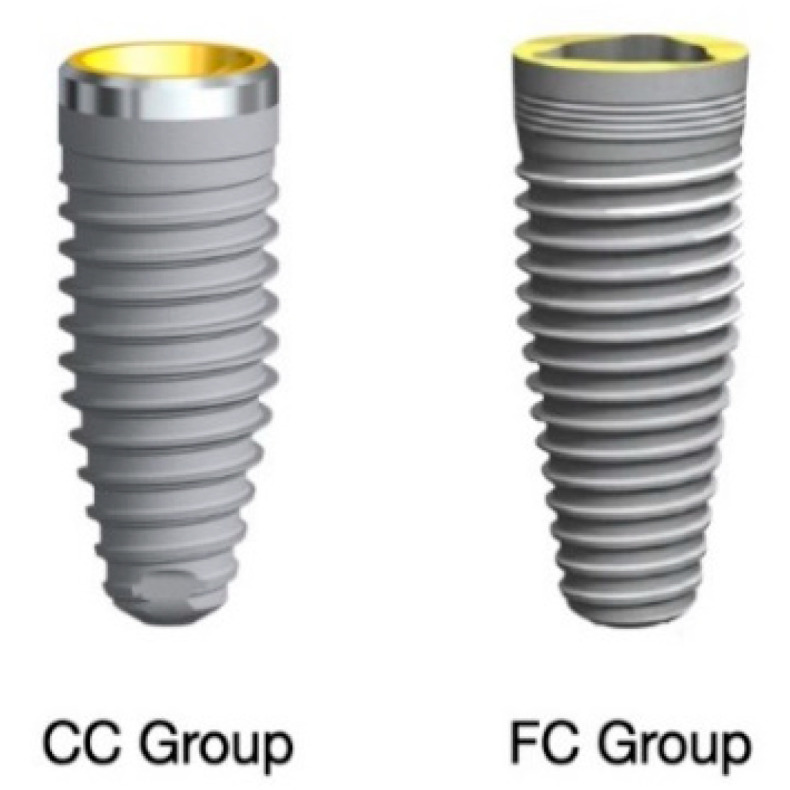
Dental implant with conical connection and partial machined collar of 0.75 mm (CC Group) and implant with internal flat-to-flat connection and groovy collar (FC Group).

**Figure 2:**
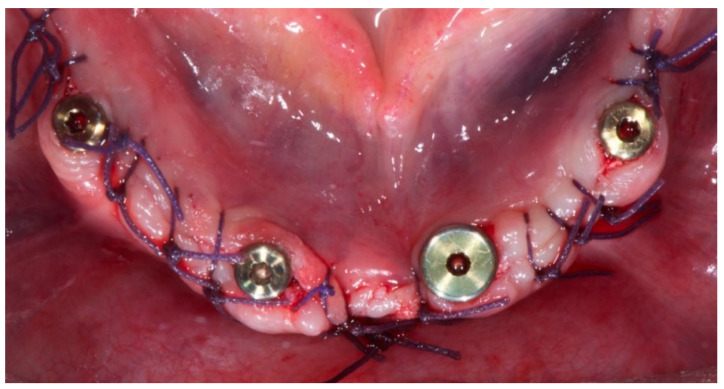
Implant placement (CC group).

**Figure 3:**
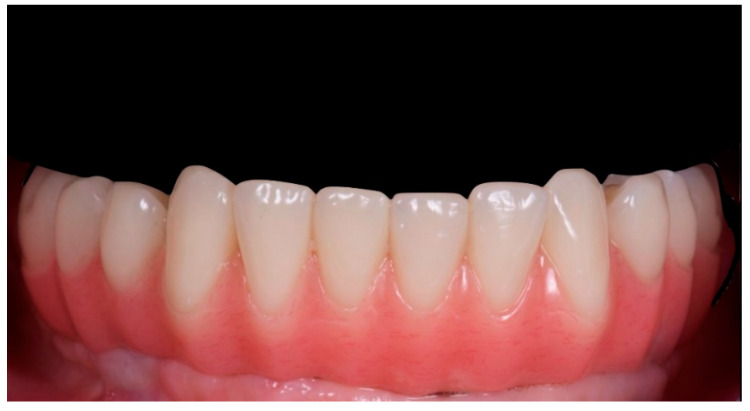
Final prosthesis at the three-year follow-up, frontal view (CC group).

**Figure 4:**
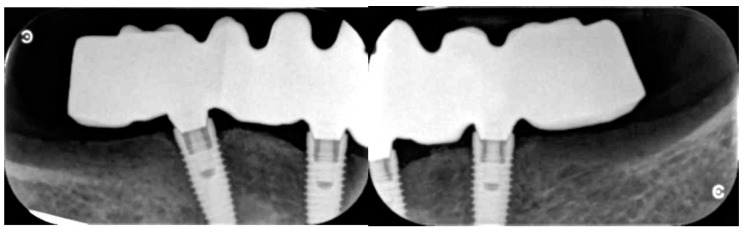
Peri-apical X-rays at the three-year follow-up (CC group).

**Figure 5:**
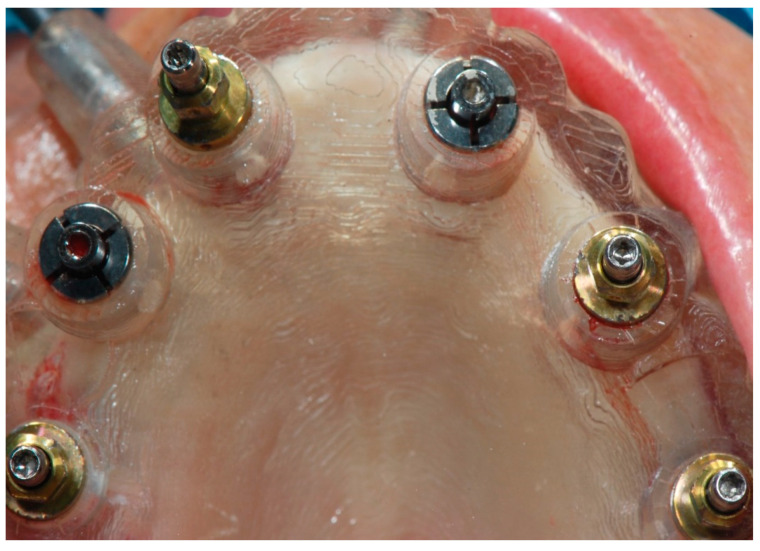
Implant placement (FC group).

**Figure 6:**
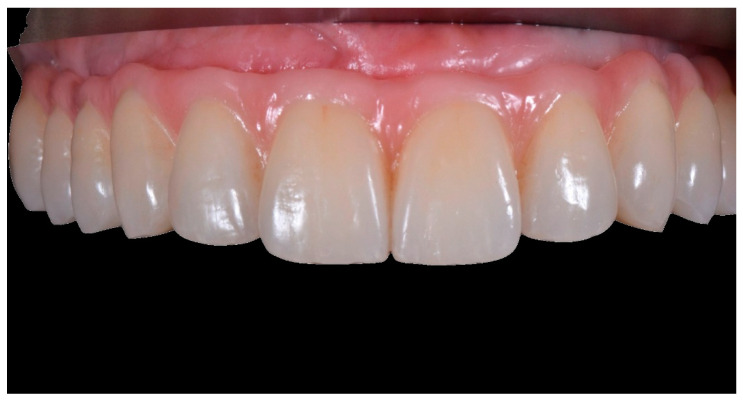
Final prosthesis at the three-year follow-up, frontal view (FC group).

**Figure 7:**
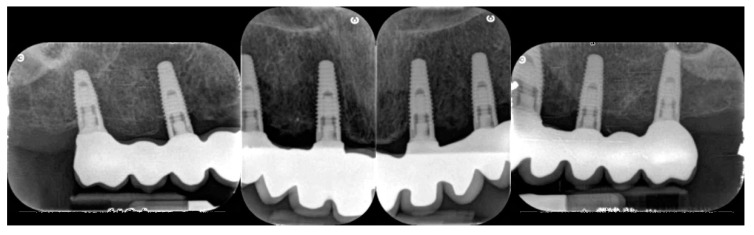
Peri-apical X-rays at the three-year follow-up (FC group).

Implants were placed according to the manufacturer instructions and previously published protocol [29,30,31,32]. The NobelReplace CC PMC implants (CC Group) and NobelReplace Tapered Groovy implants (FC Group) were fabricated with the same body-design (tapered) and type of surface (TiUnite, Nobel Biocare), but different crestal module. Implants in CC group presented internal conical connection and partially machined collar of 0.75 mm (Figure 1, Figure 2, Figure 3 and Figure 4), while implants in the FC group are characterized by internal flat-to-flat trilobe connection and rough collar surface (groovy collar, Figure 5, Figure 6 and Figure 7).

### Outcomes Measures

- Implant failure was defined as mobility, infection, fracture, and/or any other mechanical or biological issue that determined implant removal.
- A prosthesis was considered a failure any time it had to be replaced;
- Any biological complications (e.g., pain, swelling, mobility, suppuration) and/or technical issues (e.g., material fractures, screw loosening) were reported during follow-up;
- Peri-implant bone levels changes were measured as the distance between mesial and distal margin of the implant neck (inserted slightly below buccal bone level) to the most coronal point where the bone looked to be in contact with implant. Mean values of mesial and distal measurements were calculated for each implant. Measurements on digital periapical radiographic images, obtained by parallel cone technique with extension cone paralleling instrument (Rinn XCP, Dentsply, Elgin, IL, USA), were taken after implant placement (baseline) and three years after final loading. All radiographs have been analyzed through a software (DFW2.8 for Windows, Soredex, Tuuka, Finland), calibrated for each image using the known distance between two implant threads’ consecutive steps. In case of an unclear image, the radiograph was taken again. A dentist, not previously involved in this study, performed every radiographic measurement.

Differences between the two groups was compared with Fisher exact probability test for dichotomous variables (implant and prostheses failures and complications) and Mann–Whitney U tests for continuous variables (peri-implant bone levels). All the analyses were conducted at the 0.05 level of significance. Implants were used as the statistical unit. Statistical analysis was performed using software (SPSS for Mac OS X v22.o, Chicago, IL, USA).

## 3. Results

Out of 158 medical records, data from 82 patients (44 women, 38 men; range 28–72 years old; average age 55.6 years) were selected according to inclusion and exclusion criteria. A total of 152 implants were inserted and divided in two groups (77 in CC group, and 75 in FC group). Treatment indications and prosthetic rehabilitations between groups are summarized in Table 1.

Three years after final loading, one implant in CC group failed, resulting in an implant survival rate of 98.7%, while not implants failed in FC group (100% survival rate). At the last follow-up, one restoration failed in CC group (98.7% of survival rate), with no failed restorations in the FC group (100% survival rate). No biological or technical complication was experienced in either group at the three-year follow-up assessment. All the patients were enrolled in a strictly maintenance program with follow-up visit every four to six months maintaining bleeding on probing and/or plaque index equal or lower than 25%, according to the inclusion/exclusion criteria of the present research. Differences in implant and prosthetic survival rates, as well as complications between groups were not statistically significant (*p* = 1.0).

One year after loading, in the CC group, the mean marginal bone level was 0.15 ± 0.08 mm (95% CI 0.12–0.18 mm). At the three-year follow-up, the mean marginal bone level was 0.37 ± 0.1 mm (95% CI 0.34–0.4 mm). In the FC group, the mean marginal bone level at baseline was 0.03 ± 0.05 mm (95% CI 0.01–0.05 mm), while at the three-year follow-up the mean marginal bone level was 0.65 ± 0.29 mm (95% CI 0.55–0.75 mm). Three years after final loading, the mean marginal bone loss was 0.22 ± 0.06 mm (95% CI 0.2–0.24 mm) in CC group, and 0.62 ± 0.30 mm (95% CI 0.52–0.62mm) in FC group. The difference was statistically significant with higher value in the FC group (0.40 ± 0.13 mm; 95% CI 0.3–0.5 mm; *p* = 0.003). All the data are summarized in Table 2.

## 4. Discussion

This study seems to find a superior radiological outcome for conical connection implants with a smooth collar of 0.75 mm, when compared with implant with flat-to-flat connection and groovy collar design. In fact, the difference in term of MBL was statistically significant (0.40 ± 0.29 mm; *p* = 0.003). On the contrary, implants and prostheses failures, as well as complications, were not statistically significant different between the two implant–abutment configurations.

Similar outcome has been found in an RCT by Pozzi and co-authors [1,2], but in this randomized controlled trial, the external connection have been compared with conical connection implants but with a different implant design. Strength of our study is the use of the same implant differing only for the groovy or machined crestal module.

The crestal module is considered an area of strategic importance for the long-term success of an implant-prosthetic rehabilitation, optimal preservation of peri-implant tissues [20,21,22,23], since it is here that most of biological and mechanical complications may occur. Biological complications are mainly characterized by the increase in micro-infiltration, gingivitis, and crestal bone loss, due to fixture-abutment interface issues. Mechanical complications, such as increase in the incidence of rotation, abutment rupture, screw loosening, and preload reduction, can also be related to poor implant components adaptation [33].

In 2015, Wang et al. [33] analyzed the success rate of platform and non-platform switching abutments in posterior healed sites, including that PS and CC connections have both been contributory to the maintenance of the peri-implant bone. Nevertheless, the magnitude of the abutment-implant platform mismatch is a relevant factor as demonstrated by previous randomized trial [27]. According to this result, positive effect on narrow diameter implants is limited.

In a recent systematic review Schmidt et al. [34], concluded that the CC implants seem to be more resistant against abutment movements, and micro gap enlargement when loaded. CC connections have higher torque loss resistance than other systems, and also, high resistance to fatigue loading and maximum bending. However, not all studies reported better performance for conical implant–abutment connections, when compared to non-conical systems, particularly in terms of reducing strain distribution around implant [35]. Hence, the implant–abutment matching design could affect stress and strain magnitudes in a bone simulator, on the contrary, implant diameter may be more effective than the type of implant, fatigue test, screw torque loss, and screw design may be a significant factor in loosening of the joint [36,37].

In both groups of the present study, implants with a TiUnite surface (Nobel Biocare) were used. According to the literature, osseointegration of dental implants with TiUnite surface (Nobel Biocare) is very well established [38,39,40]. However, authors have demonstrated that once the surface becomes exposed the oral environment, the surface may negatively impact peri-implant health and stability, suggesting that the TiUnite surface (Nobel Biocare) may negatively affect implant success, cumulative survival rate, frequency of biologic complications [41,42].

The main limitations of this study are its retrospective design, and the relative short-term follow-up. Although different treatment options and prosthetic rehabilitations were considered, there was not statistically significant imbalance between groups. Nevertheless, despite the homogeneous samples compared and the same implant adopted, different only for the connection type, some limits are still present in this study, such as its retrospective nature. Using a paralleling technique with a Rinn device to assess the peri-implant bone loss would not be considered standardizing radiographs due to small alterations of the tube position that can influence the data recordings [42,43,44]. Moreover, two different crestal modules have been used: a micro-roughed one which hypothetically should favor osseointegration, and one machined in 0.75 coronal millimeters. Despite this, implants with a machined collar showed lower levels of marginal bone resorption. This fact may support the importance of centripetally moving the abutment–implant connection to contribute to reducing marginal bone resorption. Obviously, it should be underlined that the same prosthetic rehabilitation has been performed in both groups, and the occlusal forces have been controlled in both groups; it is well known that occlusal forces, and the type of prosthetic rehabilitation could affect the marginal bone loss [14].

## 5. Conclusions

With the limitation of this retrospective comparative study, it can be concluded that better results in preserving the marginal bone can be expected using implants with conical connection and partial machined collar when compared with implants with flat connection and groovy collar. Further studies with higher sample size are needed.

## Figures and Tables

**Table 1:** Treatment indications and prosthetic rehabilitations.

	CC Group (*n* = 77)	FC Group (*n* = 75)	*p* Value
Partial	26	24	0.864
Single	6	5	0.791
Edentoulus	45	46	0.718
Guided surgery	47	47	0.841
Flapless Surgery	30	28	0.841

**Table 2:** Mean marginal bone levels and difference between groups.

	Mean ± SD (95% CI) (mm)		Difference	*p* Value
	Baseline	Three-Year FU		
CC Group	0.15 ± 0.08 (0.12–0.18)	0.37 ± 0.1 (0.34–0.4)	0.22 mm ± 0.06 (0.2–0.24)	0.000
FC Group	0.03 ± 0.05 (0.01–0.05)	0.65 ± 0.29 (0.55–0.75)	0.62 ± 0.30 (0.52–0.72)	0.000
			0.40 ± 0.29 (0.3–0.5)	
*p* Value	0.000	0.000	0.003	

N = 77 in the CC group and N = 75 in the FC group; FU = Follow-Up.
